# Cement augmentation of the proximal humerus internal locking system in elderly patients: a multicenter randomized controlled trial

**DOI:** 10.1007/s00402-019-03142-6

**Published:** 2019-02-25

**Authors:** Clemens Hengg, Stefaan Nijs, Tim Klopfer, Martin Jaeger, Andreas Platz, Tim Pohlemann, Reto Babst, Jochen Franke, Franz Kralinger

**Affiliations:** 1 Department for Trauma Surgery, A.ö. Landeskrankenhaus - Universitätskliniken Innsbruck, Anichstrasse 35, 6020 Innsbruck, Austria; 2 Department of Traumatology, UZ Leuven, Herestraat 49, 3000 Leuven, Belgium; 3 Department for Traumatology and Reconstructive Surgery, BG Trauma Center, Eberhard Karls University Tübingen, Schnarrenbergstrasse 95, 72076 Tübingen, Germany; 4 Department of Orthopedics and Trauma Surgery, Medical Center-Albert-Ludwigs-University of Freiburg, Hugstetter Strasse 55, 79106 Freiburg, Germany; 5 Department of Traumatology, City Hospital Triemli Zurich, Birmensdorferstrasse 497, 8063 Zurich, Switzerland; 6 Department of Trauma-, Hand- and Reconstructive Surgery, Saarland University, Kirrberger Strasse, Bldng. 57, 66421 Homburg, Germany; 7 Department of Orthopedics and Traumatology, Cantonal Hospital Lucerne, Spitalstrasse, 6000 Lucerne 16, Switzerland; 8 Clinic for Trauma and Orthopaedic Surgery, BG Trauma Center Ludwigshafen, Ludwig-Guttmann-Strasse 13, 67071 Ludwigshafen, Germany; 9 Medical University of Vienna, Spitalgasse 23, 1090 Vienna, Austria; 10 Trauma and Sports Department, Teaching Hospital Medical University of Vienna, Wilhelminenspital, Montlearstr. 37, 1160 Vienna, Austria

**Keywords:** Proximal humerus fracture, Proximal humerus internal locking system, PHILOS, Augmentation, Osteoporosis, Multicenter randomized trial

## Abstract

**Introduction:**

Cement augmentation of the proximal humerus internal locking system (PHILOS) screws might reduce complication rates in osteoporotic bones. This study compared the risk of mechanical failure during the first year after PHILOS™ treatment of proximal humerus fractures (PHF) without (control group) and with (augmented group) screw augmentation. Secondary objectives were to report shoulder functions, quality of life (QoL), adverse events (AEs), and reoperation rates.

**Materials and methods:**

This multicenter randomized trial enrolled patients aged ≥ 65 years with displaced/unstable PHF from eight European centers. Randomization was performed during surgery through sealed opaque envelopes. Mechanical failures were assessed by two independent reviewers via radiographs, shoulder function by Quick DASH, SPADI, and Constant Murley scores, and QoL by EQ-5D. Follow-ups were planned at postoperative 6 weeks, 3, 6, and 12 months.

**Results:**

The preliminary analysis of 6-week radiographs of the first 59 enrolled patients suggested a mechanical failure rate lower than expected and the difference between groups was too small to be detected by the planned sample size of 144. The trial was prematurely terminated after 67 patients had been enrolled: 34 (27 eligible) in the control group and 33 (29 eligible) in the augmented group. Follow-ups were performed as planned. Nine patients had mechanical failures and the failure rates (95% CI) were: augmented group, 16.1% (5.5; 33.7); control group, 14.8% (4.2; 33.7); the relative risk (95% CI) for the augmented group was 1.09 (0.32; 3.65) compared to the control group (*p* = 1.000). No statistically significant differences in shoulder function, QoL, and AEs were observed between study groups at 1 year. Nine patients (15.8%) underwent a revision.

**Conclusions:**

Due to premature termination, the study was underpowered. A larger study will be necessary to determine if cement augmentation lowers the risk of mechanical failure rate.

## Introduction

Complex proximal humerus fractures (PHF) often occur in older adults, especially in women, and are oftentimes osteoporotic in nature [1]. The main goal in treating displaced fractures or fracture dislocations is to achieve good clinical shoulder function with no pain via restoration of the proximal humeral anatomy—a goal best achieved by open reduction and internal fixation (ORIF) together with the use of locking plates [2–9]. The proximal humerus internal locking system (PHILOS) has gained wide acceptance and shown good functional outcomes (e.g., assessed using the Constant Murley Score) [10–12]. Nevertheless, even when using PHILOS, the reported complication rates and reoperation frequencies remain high [3, 8, 13–16]. Many of the reported complications were mechanical failures such as loss of reduction and secondary screw perforation, which are believed to be associated with poor anchorage in osteoporotic bone [17, 18]. Other risk factors include aging, low local bone mineral density (BMD), lack of anatomical reduction, poor restoration of medial cortical support, fracture severity (3- and 4-part fractures), and a varus impacted fracture [3, 4, 7–9, 14, 17–20].

With the aging of the global population, the incidence of osteoporotic fractures is expected to increase. Reduced bone quality presents a challenge to orthopedic surgeries, where complications such as postoperative nonunion, screw cutout, and implant migration, adversely affect patient outcomes [21]. Both Egol et al. and Owsley et al. suggested that age influences the rate of screw cutout in PHF patients [4, 22]. Kralinger et al. reported a mechanical failure rate of 35% in a prospective multicenter cohort study of 150 patients with displaced PHF fixed with PHILOS [23].

Biomechanical tests of the proximal humerus showed that augmentation of screw tips with polymethylmethacrylate (PMMA) cement improved the mechanical properties of the bone-implant complex, especially in low mineral density bones [24–26]. To date, there has been little clinical evidence supporting the benefit of PMMA augmentation of screw tips [27–29].

The primary objective of the current study was to compare the mechanical failure rates of PHILOS treatment without and with screw augmentation in elderly PHF patients at 1-year post-surgery. Secondary objectives were to compare the study groups regarding shoulder functions, quality of life (QoL), adverse events (AEs), and reoperation rates.

## Patients and methods

### Study design and setting

The present study was a multicenter randomized controlled trial (ClinicalTrials.gov identifier: NCT01847508), with a follow-up period of 1 year after the initial treatment. Patients were enrolled from eight European study centers between January 2014 and April 2016. The last follow-up examination took place in April 2017. Enrolled patients were randomized into two groups. The control group received PHILOS™ (DePuy Synthes, Oberdorf, Switzerland) without augmentation and the augmented group received PHILOS™ Augmentation (PHILOS™ with screw augmentation using Traumacem V + Cement Kit, DePuy Synthes, Oberdorf, Switzerland). Randomization was stratified for each participating center and took place during surgery via opaque sealed envelopes after the fracture reduction was achieved and cannulated locking screws were inserted into the proximal part of the PHILOS™ plate. Three block sizes were used, with the first block always consisting of six patients and the subsequent blocks of either two or four patients, chosen at random. To maintain allocation concealment, the pattern of the blocks was kept confidential. Patients allocated to the augmented group who failed the leakage test (i.e., with zero or one screw hole suitable for augmentation) received the control treatment and were kept in the study.

The site staff entered all the data into a web-based Electronic Data Capture system, REDCap [30], hosted at the AO Foundation.

### Patients

Patients aged 65 years and older, diagnosed radiographically with an acute (≤ 10 days), closed, displaced or unstable 3- or 4-part PHF sustained after low-energy trauma, and scheduled for primary fracture treatment with a PHILOS™ plate were included.

Patients with bilateral or previous PHF, cuff-arthropathy on either side, a splitting fracture or an impression fracture of the humeral head, or associated nerve or vessel injuries were excluded. Any known clotting disorders, severe cardiac and/or pulmonary insufficiencies, severe systemic diseases classified as American Society of Anesthesiologists (ASA) class IV to VI, or not medically managed severe systemic diseases classified as ASA class III were also grounds for exclusion. Furthermore, patients with known hypersensitivity or allergy to any of the components of the Traumacem V + Cement Kit were excluded. Patients were also excluded from this study if they were prisoners, had a recent history of substance abuse (i.e., excessive recreational drugs and/or alcohol consumption) that would preclude reliable assessment, or had participated in any other medical device or medicinal product study within the previous month that could possibly influence the results of the present study.

In addition, patients were excluded before randomization if they received implants other than PHILOS or PHILOS screw augmentation.

### Study treatment

Surgical treatment was performed as described in the surgical technique guides for PHILOS™ and PHILOS™ Augmentation with the use of a deltopectoral approach [31, 32]. Leakage tests were performed in the augmented group by applying a contrast dye to each screw intended to be augmented. If no leakage into the shoulder joint was detected (i.e., negative leakage test), an injection of cement (≤ 0.5 ml) was performed under image intensifier control [24]. In case of leakage into the joint (positive leakage test), an alternative screw was selected for augmentation. To ensure a relatively homogenous effect of augmentation, each patient in the augmented group must have 2–4 screws augmented.

### Objectives and endpoints

Outcomes were evaluated at baseline (before surgery), during surgery, and at 6 weeks, 3 months, 6 months, and 12 months after surgery.

Relevant baseline data including BMD (measured by CT in the contralateral humeral head) [33] and the comorbidity status (assessed according to the Charlson Comorbidity Index) [34, 35] were assessed before surgery and randomization.

The primary endpoint was the occurrence of mechanical failure during the first year after treatment. Mechanical failures were defined as loss of reduction (≥ 15° increase of varus malposition and a relative change of ≥ 5 mm of the greater or lesser tuberosity), humeral head impaction (≥ 5 mm difference in the outer plate edge and tangent of the humeral head), screw/plate loosening (any outward movement of screw position), and secondary screw perforation (perforation of 1 or more screws through the humeral head). The final assessment of mechanical failure for each patient was done after the last follow-up visit by two experienced independent reviewers. Radiographs were taken postoperatively and at each follow-up visit. Follow-up radiographs from each patient were compared with their postoperative radiographs to determine whether mechanical failures had occurred. Disagreement between the reviewers was resolved by consensus. The mechanical failure risk within the first year after treatment was compared between the treatment groups.

Secondary endpoints: Shoulder function was measured by the Quick Disabilities of the Arm, Shoulder and Hand (DASH) measure, the Shoulder Pain and Disability Index (SPADI), and the Constant Murley score [36–43]. Quality of life (QoL) was measured using the EuroQol-5D (EQ-5D) questionnaire. Local and general AEs were recorded for both groups. For the augmented group, the number of incidences with direct contrast fluid leakage into the joint and augmentation-related AEs was also recorded.

Quick DASH, SPADI, and QoL were evaluated at each visit; baseline assessment referred to the pre-injury status. Constant Murley score was assessed at 3, 6, and 12 months after surgery. AEs were recorded from surgery until the end of the final follow-up.

### Postoperative care

All the postoperative treatments were done according to the standard of care at the investigational sites. The end of each patient’s postoperative immobilization with a shoulder sling and the start of active range of motion (ROM) were documented.

### Statistical analysis/sample size

The clinical settings and the definitions of mechanical failures of previously reported PHF studies have been quite heterogeneous. The sample size for the current study was calculated based on an earlier prospective multicenter study with a similar design that reported a mechanical failure rate of 35% [23] in 150 patients aged 50 years or older. Since the current study was designed to include an older patient population (≥ 65 years), the mechanical failure rate for the control group was assumed to be slightly higher and estimated at 40%. Augmentation was hypothesized to reduce the risk of mechanical failure rate to 15%. With a power of 80%, a significance level of 5%, a 1-year follow-up rate of ≥ 80%, and equal treatment group sizes, the sample size calculation resulted in 144 patients (72 per group).

### Preliminary analysis

Due to slow recruitment, a preliminary mechanical failure analysis was done on the first 59 patients. All 6-week follow-up images collected were evaluated by two independent reviewers not involved in the recruitment of patients. The mechanical failure rates were calculated for each study group by an independent statistician. The results showed that the mechanical failure rates of both treatment groups were drastically lower than expected and were very similar to each other. The original sample size was underpowered to detect such a small difference between the two groups; therefore, the study was prematurely terminated after 67 patients had been enrolled. Follow-ups were conducted as planned.

Both intention to treat (ITT) and per protocol (PP) analyses were performed for all outcome parameters. Simple summary statistics were produced for all the outcomes. Comparison of treatment groups was tested at the two-sided 5% significance level. For the calculation of the risk of mechanical failure, patients with incomplete follow-up and without radiographic mechanical failures were excluded. The risk of mechanical failure was calculated as follows:

$${\text{Number of patients experiencing at least 1 mechanical failure}}/\left( {{\text{Number of patients completed}} {\text{ 1-year follow-up}}+{\text{ Number of patients with incomplete }}{\text{1-year follow-up but had earlier radiographic mechanical failures}}} \right)$$

The Clopper–Pearson method was used to calculate the 95% confidence intervals (CI) for risk of mechanical failure in the two treatment groups. A two-sided Fisher’s exact test was used to compare the risk of mechanical failure between the control and the augmented groups. Treatment effects were expressed as relative risks along with their 95% CI.

Functional outcomes (Constant Murley score, Quick DASH, and SPADI) and QoL (EQ-5D index and EQ-5D VAS) were analyzed using mixed effects models for repeated measures. The models included the fixed categorical effects of treatment, visit and treatment-by-visit interaction, as well as a random effect for study center. An unstructured covariance matrix was used to model the within-patient errors. Models were fitted using restricted maximum likelihood estimation. Significance tests were based on least-squares means.

Time to end of immobilization and time to start with active ROM were analyzed using Kaplan–Meier plots and log-rank tests.

Differences in AE rates between the treatment groups were tested using Fisher’s exact test.

## Results

### Patient disposition

**Fig. 1:**
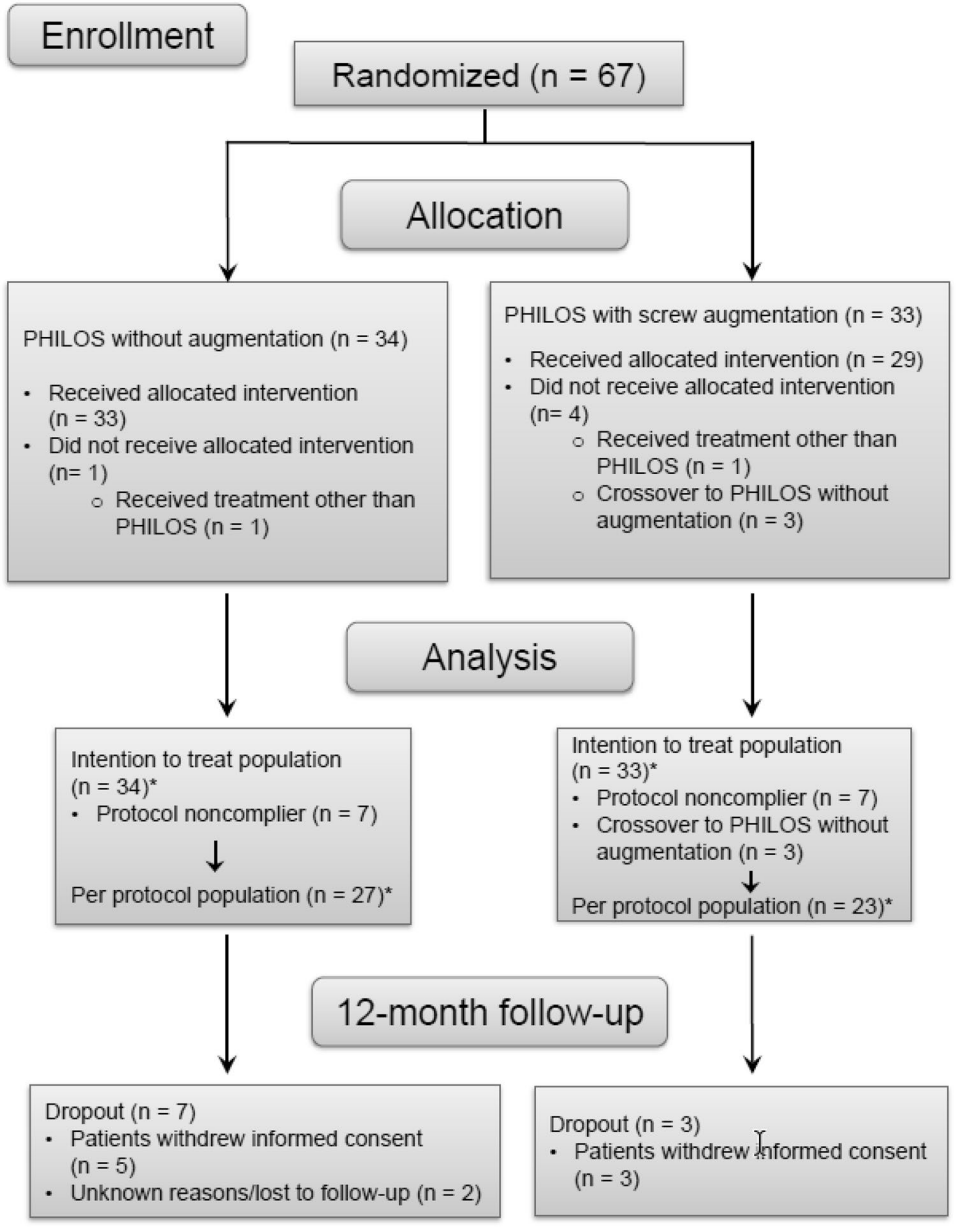
CONSORT flowchart for patient recruitment

The preliminary analysis showed a failure rate of 20% for the control group instead of the expected 40%, and 12.9% for the augmented group. Given that the assumptions for the sample size calculation were wrong and the recruitment was very slow, the study was prematurely terminated after 67 patients had given informed consent and were randomized. The control group included 34 patients, of these 27 were eligible; the augmented group had 33 patients, of these 29 were eligible, Fig. 1.

Eleven patients were determined to be ineligible after randomization but were kept in the study. The reasons for ineligibility included: five patients had two-part fractures, one had a two-part fracture and the injury was older than 10 days, two had fractures older than 10 days, one had associated nerve/vessel injury, and two received implants other than PHILOS. Three more patients had protocol violation due to the following reasons: having more than four screws augmented, not having a leakage test performed before augmentation, and/or receiving screw augmentation despite joint perforation. In total, the treatment of 14 patients did not conform to the original protocol. Three patients from the augmented group crossed over to the control group due to positive leakage tests, resulting in 50 patients included for PP analysis.

The follow-up rate at 1-year was 85.1%: ten patients (seven from the control and three from the augmented group) dropped out. The reasons for dropping out were either unknown (two patients) or withdrawal of consent (eight patients) (Fig. 1).

### Demographics and description of study population

Of the 67 randomized patients, 55 (82.1%) were women. The mean age was 76.8 ± 6.8 (Table 1). All patients except for two from the augmented group sustained their injury due to a fall. Two patients from the augmented group smoked (three and eight cigarettes a day). All the patients were Caucasians and lived at home. The mean BMD was 87.2 ± 20.1 mg/cm^3^. The median (range) Charlson Comorbidity score was 0.0 points (0.0–3.0).

**Table 1:** Summary of patient demographics and clinical characteristics (ITT analysis)

Characteristic	PHILOS without augmentation *N* = 34	PHILOS with screw augmentation *N* = 33	Total *N* = 67
Gender, *n* (%)	34	33	67
Female	29 (85.3)	26 (78.8)	55 (82.1)
Male	5 (14.7)	7 (21.2)	12 (17.9)
Age (years)			
*n*	34	33	67
Mean (SD)	76.1 (6.2)	77.5 (7.4)	76.8 (6.8)
Median (min; max)	76.0 (65.0; 90.0)	80.0 (65.0; 92.0)	77.0 (65.0; 92.0)
Smoker, *n* (%)	34	33	67
No	34 (100.0)	31 (93.9)	65 (97.0)
Yes	0 (0.0)	2 (6.1)	2 (3.0)
Bone mineral density mean (mg/cm^3^)			
*n*	28	27	55
Mean (SD)	88.1 (21.8)	86.1 (18.6)	87.2 (20.1)
Median (min; max)	86.7 (42.7; 148.4)	82.0 (51.1; 128.9)	84.5 (42.7; 148.4)
Mechanism of injury, *n* (%)	34	33	67
Fall	34 (100.0)	31 (93.9)	65 (97.0)
Other	0 (0.0)	2 (6.1)	2 (3.0)
Neer classification, *n* (%)	34	33	67
Anatomical neck two-part	1 (2.9)	0 (0.0)	1 (1.5)
Surgical neck two-part	3 (8.8)	1 (3.0)	4 (6.0)
Greater tuberosity two-part	1 (2.9)	0 (0.0)	1 (1.5)
Greater tuberosity three-part	15 (44.1)	15 (45.5)	30 (44.8)
Lesser tuberosity two-part	0 (0.0)	0 (0.0)	0 (0.0)
Lesser tuberosity three-part	2 (5.9)	1 (3.0)	3 (4.5)
Four-part	9 (26.5)	15 (45.5)	24 (35.8)
Anterior two-part	0 (0.0)	0 (0.0)	0 (0.0)
Anterior three-part	0 (0.0)	0 (0.0)	0 (0.0)
Anterior four-part	0 (0.0)	0 (0.0)	0 (0.0)
Anterior articular surface	0 (0.0)	0 (0.0)	0 (0.0)
Posterior two-part	0 (0.0)	0 (0.0)	0 (0.0)
Posterior three-part	0 (0.0)	0 (0.0)	0 (0.0)
Posterior four-part	3 (8.8)	1 (3.0)	4 (6.0)
Posterior articular surface	0 (0.0)	0 (0.0)	0 (0.0)
Head-splitting articular surface	0 (0.0)	0 (0.0)	0 (0.0)
Charlson comorbidity index^a^			
*n*	34	33	67
Mean (SD)	0.6 (0.9)	0.5 (0.8)	0.6 (0.8)
Median (min; max)	0.0 (0.0; 3.0)	0.0 (0.0; 2.0)	0.0 (0.0; 3.0)

*ITT* intention to treat, *PHILOS* Proximal humerus internal locking system

^a^The minimum possible score is 0 and the maximum possible score is 29. A higher score indicates a greater burden of comorbid conditions

Based on the fracture classification of Neer et al. [44], 30 patients (44.8%) sustained greater tuberosity three-part fractures and 24 (35.8%) had greater tuberosity/lesser tuberosity four-part fractures. The augmented group had more four-part fractures (45.5%) than the control group (26.5%) (Table 1).

### Primary endpoint

In total, nine patients (Table 2, ITT analysis) had mechanical failures within the first year after treatment. All the patients suffered loss of reduction and some had additional mechanical failures such as humeral head impaction (four patients), screw/plate loosening (one patient), and secondary screw perforation (five patients). No statistically significant differences in the occurrence of mechanical failures were found between the two study groups, neither in the ITT nor the PP analysis (*p* = 1.000 and 0.694, respectively, Table 2). According to the ITT analysis, the relative risk (95% CI) of mechanical failure in the augmented group was 1.09 (0.32; 3.65) compared to the control and 1.45 (0.37; 5.79) according to the PP analysis (Table 2). Overall, the number of patients suffering mechanical failures was similar between the two study groups.

**Table 2:** Mechanical failure occurrence within 1 year after surgery according to radiological review

	PHILOS without augmentation		PHILOS with screw augmentation		Relative risk^c^	*p* value^e^
	*n*/*N*	% (95% CI^b^)	*n*/*N*	% (95% CI^b^)	(95% CI^d^)	
Intention to treat analysis^a^	4/27	14.8 (4.2; 33.7)	5/31	16.1 (5.5; 33.7)	1.09 (0.32; 3.65)	1.000
Per protocol analysis^a^	3/24	12.5 (2.7; 32.4)	4/22	18.2 (5.2; 40.3)	1.45 (0.37; 5.79)	0.694

*PHILOS* Proximal humerus internal locking system

^a^Patients without radiographs (in at least two planes) for the 1-year follow-up were counted as having missing values, unless they had a radiographically assessed mechanical failure at an earlier time point

^b^Confidence intervals for percentages were calculated using the Clopper–Pearson method

^c^Relative risk comparing PHILOS with augmentation against PHILOS without augmentation

^d^Confidence interval was calculated using the Wald method

^e^Fisher’s exact test

### Secondary endpoints

#### Shoulder function

No statistically significant differences were detected in the Constant Murley scores (affected shoulder and relative score) between the study groups according to both the ITT and the PP analysis (Table 3). Although baseline Constant Murley scores were not available, the relative Constant Murley scores determined that patients in the control group had reached 78.7% (95% CI 69.0; 88.5%) and in the augmented group, 79.1% (95% CI 69.9; 88.3%), at 12 months after surgery according to the ITT analysis. According to the PP analysis, these were: control group, 83.1% (95% CI 73.0; 93.1%); augmented group, 85.5% (95% CI 74.6; 96.4%).

**Table 3:** Constant Murley Score (affected shoulder) and relative Constant Murley score over the course of follow-up, mixed effects model analysis

Intention to treat analysis	*n*	PHILOS without augmentation *N* = 34 Mean (95% CI)	*n*	PHILOS with augmentation *N* = 33 Mean (95% CI)	*p* value^c^
Constant Murley score^a^					
3 months	23	45.7 (38.2; 53.3)	20	40.5 (32.6; 48.4)	0.315
6 months	23	58.7 (51.4; 65.9)	22	55.6 (48.4; 62.8)	0.530
12 months	23	66.6 (58.7; 74.6)	27	64.4 (56.8; 71.9)	0.665
Relative Constant Murley score^b^					
3 months	21	57.0 (46.7; 67.2)	19	53.4 (43.1; 63.8)	0.612
6 months	23	71.7 (62.5; 80.9)	22	69.6 (60.6; 78.6)	0.726
12 months	23	78.7 (69.0; 88.5)	27	79.1 (69.9; 88.3)	0.954

Per protocol analysis	*n*	PHILOS without augmentation *N* = 27 Mean (95% CI)	*n*	PHILOS with augmentation *N* = 23 Mean (95% CI)	*p* value^c^
Constant Murley score^a^					
3 months	17	45.0 (36.3; 53.7)	12	45.8 (35.6; 56.0)	0.900
6 months	16	60.7 (53.4; 68.1)	15	55.6 (47.7; 63.6)	0.342
12 months	15	69.8 (62.5; 77.2)	12	69.9 (61.7; 78.0)	0.996
Relative Constant Murley score^b^					
3 months	16	54.4 (42.2; 66.6)	12	57.1 (43.8; 70.5)	0.750
6 months	16	74.3 (64.1; 84.6)	15	69.8 (59.2; 80.5)	0.510
12 months	15	83.1 (73.0; 93.1)	12	85.5 (74.6; 96.4)	0.727

Results are from a mixed effects model for repeated measures with an unstructured covariance (patient level) and a random center effect

*PHILOS* Proximal humerus internal locking system

^a^The Constant Murley score assesses shoulder function and pain in patients after shoulder injuries and various shoulder treatments. It ranges from 2 (worst) to 100 (best)

^b^Operated compared to contralateral shoulder, in %

^c^*p* values comparing mean values between the treatment groups at the specified time points

According to the ITT analyses, no statistically significant differences between the treatment groups were found in the Quick DASH and SPADI scores at any time points except for Quick DASH at 3 months where patients in the control group had better scores than the augmented group (Table 4). The PP analyses, however, showed that patients in the augmented group scored significantly worse than the control group in the Quick DASH evaluation at baseline [mean (95% CI) = 11.3 (6.6; 15.9) versus 4.9 (0.5; 9.2), respectively, *p* = 0.050], 6 weeks [mean (95% CI) = 56.8 (49.6; 64.0) versus 45.9 (38.7; 53.1), respectively, *p* = 0.037], and 3 months [mean (95% CI) = 44.0 (35.0; 53.0) versus 31.0 (22.7; 39.3), respectively, *p* = 0.038] (Table 5). By 6 months and 1 year after surgery, the augmented group still had higher mean scores, but the differences were not statistically significant. Differences between treatment groups in SPADI scores were not statistically significant according to the PP analyses.

**Table 4:** Functional outcomes and quality of life assessments over the course of follow-up, ITT, mixed effects model analysis

	*n*	PHILOS without augmentation (*N* = 34)			*n*	PHILOS with augmentation (*N* = 33)			*p* value^f^
		Mean (95% CI)	Change (95% CI)	*p* value^e^		Mean (95% CI)	Change (95% CI)	*p* value^e^	
Quick DASH^a^									
Baseline	33	6.0 (2.1; 9.9)			33	9.3 (5.3; 13.2)			0.242
6 weeks	31	49.0 (42.4; 55.7)	43.0 (35.9; 50.1)	< 0.001	30	55.7 (49.0; 62.4)	46.4 (39.3; 53.5)	< 0.001	0.164
3 months	29	32.9 (25.5; 40.2)	26.9 (19.4; 34.3)	< 0.001	27	44.8 (37.4; 52.2)	35.5 (28.1; 43.0)	< 0.001	0.025
6 months	29	25.9 (17.7; 34.0)	19.9 (11.8; 27.9)	< 0.001	28	30.7 (22.6; 38.8)	21.4 (13.4; 29.4)	< 0.001	0.405
12 months	26	20.9 (12.0; 29.8)	14.9 (6.3; 23.5)	< 0.001	29	24.1 (15.5; 32.8)	14.8 (6.5; 23.2)	< 0.001	0.607
SPADI^b^									
Baseline	33	3.8 (− 1.1; 8.8)			33	6.4 (1.4; 11.3)			0.371
6 weeks	31	53.0 (44.1; 61.9)	49.2 (41.8; 56.6)	< 0.001	29	59.3 (50.3; 68.3)	52.9 (45.4; 60.5)	< 0.001	0.299
3 months	30	33.8 (25.1; 42.5)	30.0 (21.5; 38.5)	< 0.001	28	41.6 (32.8; 50.4)	35.2 (26.7; 43.8)	< 0.001	0.195
6 months	29	25.8 (16.8; 34.9)	22.0 (12.8; 31.2)	< 0.001	29	27.7 (18.8; 36.7)	21.4 (12.2; 30.5)	< 0.001	0.757
12 months	27	18.7 (9.4; 28.1)	14.9 (5.1; 24.7)	0.004	30	22.4 (13.3; 31.5)	16.0 (6.4; 25.6)	0.002	0.568
EQ-5D index^c^									
Baseline	33	0.96 (0.89; 1.02)			33	0.94 (0.88; 1.00)			0.649
6 weeks	30	0.79 (0.70; 0.87)	− 0.17 (− 0.26; − 0.08)	< 0.001	31	0.73 (0.64; 0.81)	− 0.21 (− 0.30; − 0.12)	< 0.001	0.271
3 months	30	0.84 (0.75; 0.93)	− 0.12 (− 0.21; − 0.02)	0.016	29	0.76 (0.67; 0.85)	− 0.18 (− 0.28; − 0.09)	< 0.001	0.171
6 months	30	0.85 (0.77; 0.93)	− 0.11 (− 0.18; − 0.03)	0.005	29	0.83 (0.74; 0.91)	− 0.11 (− 0.19; − 0.04)	0.003	0.658
12 months	27	0.90 (0.82; 0.98)	− 0.06 (− 0.14; 0.01)	0.112	30	0.85 (0.77; 0.93)	− 0.09 (− 0.16; − 0.01)	0.020	0.400
EQ-5D VAS health state^d^									
Baseline	32	83.7 (77.2; 90.3)			32	81.3 (74.7; 87.8)			0.460
6 weeks	31	72.0 (64.8; 79.1)	− 11.8 (− 18.1; − 5.5)	< 0.001	31	67.1 (60.0; 74.2)	− 14.1 (− 20.3; − 7.9)	< 0.001	0.224
3 months	30	78.1 (71.3; 85.0)	− 5.6 (− 11.7; 0.5)	0.073	29	71.4 (64.6; 78.3)	− 9.8 (− 15.9; − 3.7)	0.002	0.070
6 months	30	76.5 (69.3; 83.7)	− 7.2 (− 14.2; − 0.3)	0.041	28	75.4 (68.2; 82.7)	− 5.8 (− 12.8; 1.2)	0.104	0.794
12 months	27	84.5 (77.6; 91.4)	0.7 (− 5.4; 6.8)	0.816	29	78.0 (71.2; 84.9)	− 3.2 (− 9.2; 2.8)	0.288	0.086

Results are from mixed effects models for repeated measures with an unstructured covariance (patient level) and a random center effect

*ITT* Intention to treat, *DASH* Disabilities of the Arm, Shoulder and Hand, *EQ-5D* EuroQol-5D, *PHILOS* Proximal humerus internal locking system, *SPADI* Shoulder Pain and Disability Index, *Control group* PHILOS without augmentation, *Augmented group* PHILOS with augmentation

^a^The Quick DASH score measures physical function and symptoms in people with any or multiple musculoskeletal disorders of the upper limb. It ranges from 0 (best) to 100 (worst)

^b^The SPADI score measures the pain and disability associated with shoulder pathology. It ranges from 0 to 100 with 0 indicating a perfect result and 100 reflecting the worst possible outcome

^c^The EQ-5D index score ranges from 0 (dead) to 1 (perfect health), although negative values are possible

^d^The EQ-5D VAS represents a question assessing patients’ health state (“How good or bad is your health state?”). It ranges from 0 “worst imaginable health state” to 100 “best imaginable health state”

^e^*p* values assessing the change from baseline within a treatment group at the specified time point

^f^*p* values assessing the differences between the treatment groups at the specified time point

**Table 5:** Functional outcomes and quality of life assessments over the course of follow-up, PP, mixed effects model analysis

	*n*	PHILOS without augmentation (*N* = 27)			*n*	PHILOS with augmentation (*N* = 23)			*p* value^f^
		Mean (95% CI)	Change (95% CI)	*p* value^e^		Mean (95% CI)	Change (95% CI)	*p* value^e^	
Quick DASH^a^									
Baseline	26	4.9 (0.5; 9.2)			23	11.3 (6.6; 15.9)			0.050
6 weeks	19	45.9 (38.7; 53.1)	41.0 (33.4; 48.7)	< 0.001	20	56.8 (49.6; 64.0)	45.5 (37.8; 53.3)	< 0.001	0.037
3 months	21	31.0 (22.7; 39.3)	26.1 (17.7; 34.6)	< 0.001	17	44.0 (35.0; 53.0)	32.8 (23.6; 41.9)	< 0.001	0.038
6 months	20	19.6 (10.9; 28.3)	14.7 (5.8; 23.6)	0.002	19	29.0 (19.8; 38.1)	17.7 (8.4; 27.0)	< 0.001	0.139
12 months	15	11.7 (1.6; 21.8)	6.8 (− 3.1; 16.8)	0.172	13	18.8 (8.0; 29.6)	7.5 (− 3.0; 18.1)	0.157	0.336
SPADI^b^									
Baseline	26	2.2 (−1.3; 5.8)			23	5.8 (2.1; 9.6)			0.117
6 weeks	19	50.8 (41.9; 59.8)	48.6 (40.3; 56.9)	< 0.001	20	56.1 (46.8; 65.3)	50.3 (41.8; 58.8)	< 0.001	0.408
3 months	22	32.0 (22.0; 41.9)	29.7 (20.3; 39.2)	< 0.001	18	41.1 (30.2; 52.0)	35.3 (25.0; 45.6)	< 0.001	0.213
6 months	20	22.4 (12.8; 32.0)	20.2 (10.5; 29.8)	< 0.001	20	26.3 (16.4; 36.3)	20.5 (10.6; 30.4)	< 0.001	0.568
12 months	16	13.1 (4.1; 22.1)	10.9 (1.9; 19.8)	0.019	14	20.6 (10.9; 30.3)	14.8 (5.2; 24.4)	0.004	0.251
EQ-5D index^c^									
Baseline	26	0.96 (0.89; 1.04)			23	0.93 (0.85; 1.01)			0.524
6 weeks	18	0.83 (0.73; 0.93)	− 0.13 (− 0.24; − 0.03)	0.017	21	0.70 (0.61; 0.80)	− 0.23 (− 0.33; −0.12)	< 0.001	0.051
3 months	22	0.85 (0.75; 0.94)	− 0.12 (− 0.22; − 0.01)	0.030	18	0.79 (0.69; 0.89)	− 0.14 (− 0.25; −0.03)	0.016	0.401
6 months	21	0.88 (0.80; 0.96)	− 0.08 (− 0.18; 0.01)	0.085	20	0.85 (0.76; 0.93)	− 0.08 (− 0.18; 0.02)	0.098	0.573
12 months	16	0.89 (0.80; 0.98)	− 0.07 (− 0.14; − 0.00)	0.044	14	0.84 (0.74; 0.94)	− 0.09 (− 0.17; −0.02)	0.014	0.411
EQ-5D VAS health state^d^									
Baseline	26	83.3 (76.1; 90.5)			23	78.5 (71.1; 86.0)			0.265
6 weeks	19	73.1 (65.2; 80.9)	− 10.2 (− 17.9; −2.5)	0.011	21	64.0 (56.2; 71.8)	− 14.5 (− 22.3; −6.8)	< 0.001	0.054
3 months	22	78.0 (70.6; 85.3)	− 5.3 (− 12.7; 2.1)	0.159	18	71.0 (63.2; 78.8)	− 7.5 (− 15.6; 0.5)	0.065	0.119
6 months	21	80.9 (73.6; 88.3)	− 2.3 (− 10.0; 5.3)	0.539	19	77.9 (70.4; 85.5)	− 0.6 (− 8.7; 7.4)	0.874	0.473
12 months	16	86.3 (78.5; 94.0)	3.0 (− 4.8; 10.8)	0.439	14	77.1 (68.9; 85.2)	− 1.5 (− 9.8; 6.8)	0.715	0.055

Results are from mixed effects models for repeated measures with an unstructured covariance (patient level) and a random center effect

*PP* Per protocol, *DASH* Disabilities of the Arm, Shoulder and Hand, *EQ-5D* EuroQol-5D, *PHILOS* Proximal humerus internal locking system, *SPADI* Shoulder Pain and Disability Index, *Control group* PHILOS without augmentation, *Augmented group* PHILOS with augmentation

^a^The Quick DASH score measures physical function and symptoms in people with any or multiple musculoskeletal disorders of the upper limb. It ranges from 0 (best) to 100 (worst)

^b^The SPADI score measure the pain and disability associated with shoulder pathology. It ranges from 0 to 100 with 0 indicating a perfect result and 100 reflecting the worst possible outcome

^c^The EQ-5D index score ranges from 0 (dead) to 1 (perfect health), although negative values are possible

^d^The EQ-5D VAS represents a question assessing patients’ health state (“How good or bad is your health state?”). It ranges from 0 “worst imaginable health state” to 100 “best imaginable health state”

^e^*p* values assessing the change from baseline within a treatment group at the specified time point

^f^*p* values assessing the differences between the treatment groups at the specified time point

Patients from both groups showed improvement in their Constant Murley, Quick DASH and SPADI scores throughout the follow-up period. However, the mean scores at 12 months were still significantly worse than the mean scores at baseline in Quick DASH according to the ITT but not the PP analysis [control group mean = 11.7 (95% CI 1.6; 21.8) at 12 months versus 4.9 (0.5; 9.2) at baseline, *p* = 0.172, and augmented group mean = 18.8 (8.0; 29.6) at 12 months versus 11.3 (6.6; 15.9) at baseline, *p* = 0.157]. Mean SPADI scores at 12 months were significantly worse than scores at baseline for both treatment groups according to both analyses (Tables 4, 5).

#### Quality of life

According to the ITT analyses, no statistical differences in the EQ-5D index nor the EQ-VAS health state scores were detected between the two treatment groups at any time point (Table 4). PP analyses also revealed no statistical differences for all time points, although the 6 weeks EQ-5D index *p* value (*p* = 0.051) and the 6 weeks and 12 months EQ-5D VAS *p* values (*p* = 0.054 and 0.055, respectively) were borderline significant (Table 5).

Compared to the baseline values, the EQ-5D index score for the control group was significantly worse at 12 months according to the PP analysis [mean (95% CI) = 0.89 (0.80; 0.98) at 12 months versus 0.96 (0.89; 1.04) at baseline, *p* = 0.044] (Table 5), but not according to the ITT analysis [mean (95% CI) = 0.90 (0.82; 0.98) at 12 months versus 0.96 (0.89; 1.02) at baseline, *p* = 0.112] (Table 4), whereas for the augmented group, the score at 12 months was significantly lower than baseline in both analyses (*p* = 0.020 and *p* = 0.014, Tables 4, 5).

No statistically significant difference in the EQ-5D VAS scores was detected in either treatment group (according to both the ITT and PP analyses) 1 year after surgery compared to baseline (Tables 4, 5).

#### Postoperative shoulder immobilization and time to active range of motion

**Fig. 2:**
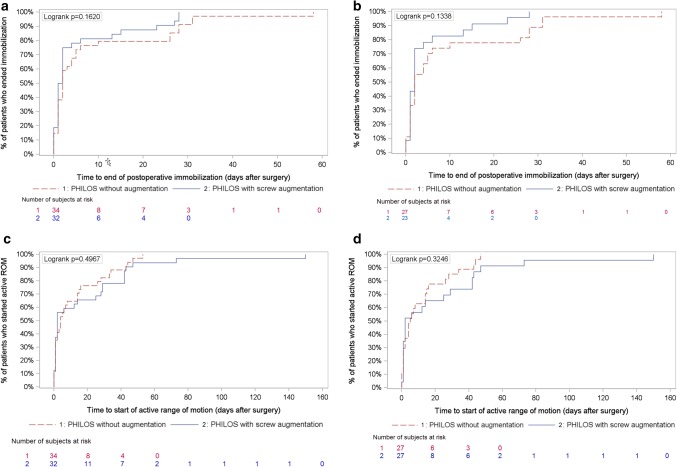
Postoperative shoulder immobilization and time to active range of motion: Kaplan–Meier analyses of number of subjects at risk. **a** Time to end of shoulder immobilization (ITT). **b** Time to end of shoulder immobilization (PP). **c** Time to start active range of motion (ITT). **d** Time to start active range of motion (PP). ITT: intention to treat analysis, PP: Per protocol analysis. Control group: PHILOS without augmentation. Augmented group: PHILOS with augmentation

The median (range) time of postoperative shoulder immobilization was 2.0 days (0 to 58) in the control group and 2.0 days (0 to 28) in the augmented group. The time to reach the end of immobilization did not differ significantly between the two groups according to ITT analyses (*p* = 0.162) and PP analyses (*p* = 0.134) (Fig. 2a, b, respectively).

Patients in the control group started with active ROM at a median (range) of 4.0 days (0–53), and in the augmented group, 2.0 days (0–150). There were no significant differences between the two groups (*p* = 0.497, ITT analyses; *p* = 0.325, PP analyses) (Fig. 2c, d, respectively).

#### Radiological outcomes

Radiological outcomes were analyzed using the safety population, defined as all patients who received the PHILOS™ treatment. Anatomical reduction was achieved in 24/35 patients (68.6%) of the control group (1 missing value due to the lack of postoperative radiograph) and in 20/29 patients (69.0%) of the augmented group; there was no statistically significant difference between the groups (*p* = 0.973). Restoration of medial support was achieved in 22/35 patients (62.9%) of the control group and in 18/29 patients (62.1%) of the augmented group, also with no difference between the groups (*p* = 0.948).

#### Safety analysis

Adverse events were also analyzed using the safety population. Fifteen patients in the control group and 12 patients in the augmented group reported at least 1 AE during the study (Table 6), resulting in an overall AE rate (95% CI) of 41.5% (29.4; 54.4). There was no statistically significant difference in the overall AE reporting rate between the control group, 41.7% (25.5; 59.2), and the augmented group, 41.4 (23.5; 61.1) (*p* = 1.000).

**Table 6:** Summary of adverse events (patient level) within 1 year after surgery (safety population)

Adverse events^a^	PHILOS without augmentation *N* = 36		PHILOS with screw augmentation *N* = 29		Total *N* = 65		*p* value^e^
	*N* ^b^	%^c^ (95% CI^d^)	*N* ^b^	%^c^ (95% CI^d^)	*N* ^b^	%^c^ (95% CI^d^)	
Any adverse events	15	41.7 (25.5; 59.2)	12	41.4 (23.5; 61.1)	27	41.5 (29.4; 54.4)	1.000
Any intraoperative adverse event	4	11.1 (3.1; 26.1)	1	3.4 (0.1; 17.8)	5	7.7 (2.5; 17.0)	0.370
Poor intraoperative fracture reduction	3	8.3 (1.8; 22.5)	0	0.0 (0.0; 11.9)	3	4.6 (1.0; 12.9)	–
Malpositioning of the plate/screw(s)	2	5.6 (0.7; 18.7)	0	0.0 (0.0; 11.9)	2	3.1 (0.4; 10.7)	–
Cement leakage into the joint	0	0.0 (0.0; 9.7)	1	3.4 (0.1; 17.8)	1	1.5 (0.0; 8.3)	–
Cement application into the wound	0	0.0 (0.0; 9.7)	0	0.0 (0.0; 11.9)	0	0.0 (0.0; 5.5)	–
Direct allergic reaction to Traumacem V + cement	0	0.0 (0.0; 9.7)	0	0.0 (0.0; 11.9)	0	0.0 (0.0; 5.5)	–
Direct allergic reaction to contrast media	0	0.0 (0.0; 9.7)	0	0.0 (0.0; 11.9)	0	0.0 (0.0; 5.5)	–
Implant failure/breakage	0	0.0 (0.0; 9.7)	0	0.0 (0.0; 11.9)	0	0.0 (0.0; 5.5)	–
Other bone/fracture-related AE	0	0.0 (0.0; 9.7)	0	0.0 (0.0; 11.9)	0	0.0 (0.0; 5.5)	–
Nerve injury	0	0.0 (0.0; 9.7)	0	0.0 (0.0; 11.9)	0	0.0 (0.0; 5.5)	–
Other AE related to soft tissue of the musculoskeletal system	0	0.0 (0.0; 9.7)	0	0.0 (0.0; 11.9)	0	0.0 (0.0; 5.5)	–
Other wound/local tissue-related AE	0	0.0 (0.0; 9.7)	0	0.0 (0.0; 11.9)	0	0.0 (0.0; 5.5)	–
Thromboembolic complications	0	0.0 (0.0; 9.7)	0	0.0 (0.0; 11.9)	0	0.0 (0.0; 5.5)	–
Renal insufficiency	0	0.0 (0.0; 9.7)	0	0.0 (0.0; 11.9)	0	0.0 (0.0; 5.5)	–
Stroke	0	0.0 (0.0; 9.7)	0	0.0 (0.0; 11.9)	0	0.0 (0.0; 5.5)	–
Sudden death	0	0.0 (0.0; 9.7)	0	0.0 (0.0; 11.9)	0	0.0 (0.0; 5.5)	–
Intraoperative hemodynamically relevant hemorrhage	0	0.0 (0.0; 9.7)	0	0.0 (0.0; 11.9)	0	0.0 (0.0; 5.5)	-
Other systemic AE	0	0.0 (0.0; 9.7)	0	0.0 (0.0; 11.9)	0	0.0 (0.0; 5.5)	–
Any postoperative adverse event	14	38.9 (23.1; 56.5)	12	41.4 (23.5; 61.1)	26	40.0 (28.0; 52.9)	1.000
Late/development of allergic reaction to Traumacem V + cement	0	0.0 (0.0; 9.7)	0	0.0 (0.0; 11.9)	0	0.0 (0.0; 5.5)	–
Late/development of allergic reaction to contrast media	0	0.0 (0.0; 9.7)	0	0.0 (0.0; 11.9)	0	0.0 (0.0; 5.5)	–
Primary/secondary screw perforation	3	8.3 (1.8; 22.5)	1	3.4 (0.1; 17.8)	4	6.2 (1.7; 15.0)	–
Screw/plate loosening	0	0.0 (0.0; 9.7)	0	0.0 (0.0; 11.9)	0	0.0 (0.0; 5.5)	–
Implant failure/breakage	0	0.0 (0.0; 9.7)	0	0.0 (0.0; 11.9)	0	0.0 (0.0; 5.5)	–
Nonunion	0	0.0 (0.0; 9.7)	0	0.0 (0.0; 11.9)	0	0.0 (0.0; 5.5)	–
Delayed union	0	0.0 (0.0; 9.7)	0	0.0 (0.0; 11.9)	0	0.0 (0.0; 5.5)	–
Malunion	0	0.0 (0.0; 9.7)	1	3.4 (0.1; 17.8)	1	1.5 (0.0; 8.3)	–
Loss of reduction	2	5.6 (0.7; 18.7)	1	3.4 (0.1; 17.8)	3	4.6 (1.0; 12.9)	–
Humeral head necrosis	2	5.6 (0.7; 18.7)	3	10.3 (2.2; 27.4)	5	7.7 (2.5; 17.0)	–
Head impaction	1	2.8 (0.1; 14.5)	0	0.0 (0.0; 11.9)	1	1.5 (0.0; 8.3)	–
Other bone/fracture-related AE	1	2.8 (0.1; 14.5)	2	6.9 (0.8; 22.8)	3	4.6 (1.0; 12.9)	–
Deep wound infection	0	0.0 (0.0; 9.7)	1	3.4 (0.1; 17.8)	1	1.5 (0.0; 8.3)	–
Impingement	2	5.6 (0.7; 18.7)	0	0.0 (0.0; 11.9)	2	3.1 (0.4; 10.7)	–
Nerve injury	0	0.0 (0.0; 9.7)	1	3.4 (0.1; 17.8)	1	1.5 (0.0; 8.3)	–
Other AE related to soft tissue of the musculoskeletal system	1	2.8 (0.1; 14.5)	2	6.9 (0.8; 22.8)	3	4.6 (1.0; 12.9)	–
Superficial wound infection	0	0.0 (0.0; 9.7)	0	0.0 (0.0; 11.9)	0	0.0 (0.0; 5.5)	–
Wound dehiscence	0	0.0 (0.0; 9.7)	0	0.0 (0.0; 11.9)	0	0.0 (0.0; 5.5)	–
Hematoma (requiring revision)	1	2.8 (0.1; 14.5)	0	0.0 (0.0; 11.9)	1	1.5 (0.0; 8.3)	–
Other wound/local tissue-related AE	0	0.0 (0.0; 9.7)	0	0.0 (0.0; 11.9)	0	0.0 (0.0; 5.5)	–
Thromboembolic complications	0	0.0 (0.0; 9.7)	0	0.0 (0.0; 11.9)	0	0.0 (0.0; 5.5)	–
Sepsis	0	0.0 (0.0; 9.7)	0	0.0 (0.0; 11.9)	0	0.0 (0.0; 5.5)	–
Pneumonia	0	0.0 (0.0; 9.7)	0	0.0 (0.0; 11.9)	0	0.0 (0.0; 5.5)	–
Renal insufficiency	0	0.0 (0.0; 9.7)	0	0.0 (0.0; 11.9)	0	0.0 (0.0; 5.5)	–
Stroke	0	0.0 (0.0; 9.7)	1	3.4 (0.1; 17.8)	1	1.5 (0.0; 8.3)	–
Sudden death	0	0.0 (0.0; 9.7)	0	0.0 (0.0; 11.9)	0	0.0 (0.0; 5.5)	–
Other systemic AE	4	11.1 (3.1; 26.1)	3	10.3 (2.2; 27.4)	7	10.8 (4.4; 20.9)	–

The safety population was defined as all patients that have received PHILOS type of plate fixation. Patients were grouped according to the actual treatment they received. The two patients that received prothesis as the primary fixation are excluded in this table. Control group: PHILOS without augmentation. Augmented group: PHILOS with augmentation

*AE* adverse event, *PHILOS* proximal humerus internal locking system

^a^Only AEs starting up to ≤ 425 days after surgery, i.e., with an onset before the upper visit window of the 1-year follow-up, were included in the table

^b^Number of patients with at least one AE. If patient experienced multiple AEs under any complication class, the patient was only counted once

^c^Estimated risk of developing at least one AE (calculated by dividing the number of patients experiencing at least one complication by the total number of patients in the corresponding treatment group. For this table, all the patients irrespective of availability of follow-up counted in the denominator)

^d^Confidence intervals for percentages were calculated using the Clopper–Pearson method

^e^Fisher’s exact test

In total, nine patients underwent revision: one patient had two revision surgeries, first due to hematoma and later, screw perforation. The remaining revision surgeries were due to humeral head necrosis (four patients), humeral head impaction (one patient), loss of reduction (one patient), infection, and malposition of plate/screw (one patient each). Three patients were revised with anatomic prothesis and two with reverse arthroplasty.

## Discussion

The present study aimed to investigate the difference in the risk of mechanical failure in elderly PHF patients treated with PHILOS™ with or without cement augmentation of the screw tips within the first year after surgery. As recruitment was slow and a preliminary analysis showed a much lower mechanical failure rate than expected with no apparent differences between the groups, the study was prematurely terminated after enrolling less than half the intended number of patients.

The study results showed no statistically significant differences in the mechanical failure rates between study groups at 1 year. Given the early termination of the recruitment, the current study was underpowered. All the estimates of treatment effect with our final sample size had a high uncertainty. The relative risks (95% CI) of having a mechanical failure for the augmented group compared to the control group were estimated at 1.09 (0.32; 3.65) in the ITT analysis and 1.45 (0.37; 5.09) in the PP analysis. Large confidence intervals (including “1”) indicate that the evidence for this study is not sufficient, and a much larger sample size would be required to show a possible difference between the use of PHILOS™ without augmentation and PHILOS™ with augmentation in proximal humerus fractures.

Neither ITT nor PP analyses showed statistically significant differences between the treatment groups in shoulder function measured with Constant Murley score, Quick DASH, and SPADI at 1 year. PP analyses with mixed effects model for the Quick DASH showed that the augmented group had statistically significant worse scores than the control group at the initial time points (baseline, 6 weeks, and 3 months after surgery). Although the mean scores for the augmented group remained worse at 6 and 12 months after surgery compared to the control group, the differences were not statistically significant. Several potential explanations exist for this difference. One possibility is the higher proportion of patients with four-part fracture in the augmented group (45.5% versus 26.5% in the control, ITT analysis), as it has been suggested that patients with four-part fractures tend to have lower functional outcomes than those with two- and three-part fractures [15]. These results could also suggest that the impact of fracture severity on shoulder function after treatment with PHILOS is only temporary since the differences were not significant by 6 months.

Overall, results showed that the injured shoulders continued to recover through the first year after surgery. Even then, shoulder function at 1 year was still worse in both treatment groups compared to baseline. In comparison to 1-year (QUICK) DASH and Constant Murley score results reported in the literature, the current patient population reached similar recovery at 1 year [4, 12, 14, 20, 45, 46]. However, direct comparison of results from different studies may be misleading due their heterogeneity. For example, in the study reported by Gracitelli et al., the patients had two- and three-part PHFs while the current patients had three- or four-part PHF [46]. According to the relative Constant Murley score results, the shoulder function outcome of the current population reached the “good” category according to the PP analysis or the high-end of the “satisfactory” category according to the ITT analysis at 12 months postoperatively [excellent (90–100%), good (80–89%), satisfactory (60–79%), and poor (< 60%)] [46, 47].

Similar to the results of shoulder function, there were no statistically significant differences between the treatment groups in QoL at 1 year after surgery (both ITT and PP analysis), although borderline significance was observed at 6 weeks in EQ-5D index scores and 6 weeks and 12 months in EQ-5D VAS scores (PP analysis).

Screw augmentation has been shown to be effective in biomechanical studies, but clinical evidence has been lacking. A recent study by Katthagen et al. comparing 24 patients treated with PHILOS screw augmentation to a historical cohort showed that screw augmentation reduced the likelihood of early loss of reduction and articular screw perforation but resulted in no differences in shoulder function scores [27]. In contrast, the current study showed no overall differences between the treatment groups. Differences between the two studies include: (1) the current study assessed loss of reduction, humeral head impaction, screw/plate loosening, and secondary screw perforation, while the previous study reported only loss of reduction and articular screw perforation. (2) The current study was a prospective study, while the previous study was retrospective with a matched historical cohort. (3) The current study had a follow-up period of 1 year, while the previous study recorded radiological results at 3 months postoperatively. Ultimately, both studies were rather small and a larger prospective study with sufficient statistical power may help resolve the differences. One surprising outcome was the much lower than expected mechanical failure rate. With the highest mean age (76.8 ± 6.8) recorded in the PHF surgery literature so far [15, 27] and a very low BMD of 87.2 ± 20 [17, 48], the current study nevertheless achieved an overall mechanical failure rate of around 15%. Although it is difficult to compare our mechanical failure rate with literature values due to the reporting heterogeneity, the current mechanical failure rate is likely to be on the lower end of what have been reported [4, 14, 20, 23, 27, 49]. In osteoporotic fracture care, with the restoration of the medial hinge and intrinsic stability through anatomical reduction, implants become a load sharing rather than a load bearing construct leading to reduced mechanical failure rates. Therefore, one possible explanation for the current low mechanical failure rate could be the improvement of osteoporotic fracture care in the past decade: better anatomical reduction through surgical means resulting in the restoration of intrinsic stability. In comparison to a previous study with a younger study population where anatomical reduction was achieved in 25.7% (9/35) of the patients and medial support achieved in 51.4% (18/35), the current study had a much higher rate of anatomical reduction (68.6% for the control group and 69% for the augmented group) and restoration of medial support (62.9% for the control group and 62.1% for the augmented group) [7]. This is consistent with the previous suggestion that the most important factor in determining good outcomes was good surgical techniques [7, 11, 14, 17, 49]. In addition, a Hawthorne effect cannot be ruled out: given the prospective study design, the surgeons may have paid more attention to surgical details contributing to the observed lower mechanical failure rate.

Although it is hard to compare the AE rate of different studies due to their heterogeneity (e.g., different reporting criterion, study design, patient population, and different surgical techniques), the currently reported AE rate of 41.7% in the control group and 41.5% in the augmented group are in the upper range of previous reports [5, 9, 10, 14, 20, 22, 49]. This could be due to the prospective nature of the study and a more comprehensive collection of AEs. Consistent to a previous report that no additional complications were noted in screw augmentation in PHF [27], in the current study, only one patient suffered an AE related to the used Traumacem V + cement, i.e., intraoperative cement leakage into the shoulder joint. After a joint arthrotomy, the patient fully recovered. No allergic reaction to the Traumacem V + cement was reported.

Currently, it is common to perform primary reversed fracture arthroplasty in three- and four-part fractures with a cutoff age of 75 years [50, 51]. For complex fractures, this procedure is performed in patients aged 65 years and up (surgeon’s observation). Our result of a low mechanical failure rate reported in both the control and the augmented groups would suggest that good outcome can be achieved in elderly complex PHF patients with low BMD treated with locking plates, and that a further lowering the age for primary reversed fracture arthroplasty may not be reasonable.

### Limitations

The major limitation of the current study is its premature termination and a much smaller patient population than the original plan. For this reason, the study did not have the statistical power to detect differences between the study groups.

### Future direction

Due to the difficulty in patient recruitment in the current study and that past RCTs have not resulted in sufficient evidence to recommend a treatment of choice, we anticipate that future RCTs are likely to encounter similar problems [52–54]. Prospective observational studies and registries are more likely to generate data from sufficiently large patient populations and are the recommended way to move the field forward. In light of the ongoing debate in the treatment protocol for complex PHF, i.e., arthroplasty versus osteosynthesis, nail versus plates, and conservative versus surgical, an observational approach also has the benefit of comparing multiple methods in one study.

## Conclusion

Due to premature termination resulting in an underpowered study, evidence was not sufficient to show any differences between the two treatment groups regarding the risk of mechanical failure. However, considering that no additional risks related to the use of cement were observed, and that low mechanical failure rate was achieved in a population with high mean age and low BMD, PHILOS™ seems a good treatment option for elderly PHF in patients. Further studies are necessary to establish the effectiveness of screw-tip cement augmentation of PHILOS.
